# First Evidence of *Hepatozoon canis* in Dogs With Clinical Signs in the North of Lima, Peru

**DOI:** 10.1155/japr/5430576

**Published:** 2025-08-21

**Authors:** Cesar Ramirez, Daphne León, Luis M. Jara

**Affiliations:** Facultad de Medicina Veterinaria y Zootecnia, Universidad Peruana Cayetano Heredia, Lima, Peru

**Keywords:** blood smear, dogs, hemopathogens, *Hepatozoon*

## Abstract

This study was aimed at detecting the presence of *Hepatozoon canis* in dogs with clinical signs in the northern area of Lima, Peru. Peripheral blood samples (*n* = 152) were collected from dogs with hematological alterations and clinical signs suggestive of hemoparasitosis. PCR was used to amplify the 18S ribosomal RNA gene of *Hepatozoon* sp. and was complemented with Sanger sequencing. Blood smears were used to identify the parasite gamonts. The frequency of *H. canis* was 15.1% (23/152) based on PCR and 0.7% (1/152) based on microscopic observation. The resulting sequences of the positive PCR amplicons (574 bp) exhibited high sequence similarity to the previously registered *H. canis* sequences in GenBank. Phylogenetic analysis revealed a similar relationship between the sequences identified in dogs from Thailand, the Philippines, Pakistan, India, and Colombia. This study presents the first evidence of *H. canis* in dogs with clinical signs in an endemic area for vector-borne diseases in Lima, Peru.

## 1. Introduction

Canine hepatozoonosis is an emerging parasitic disease with global distribution in several South American countries [1]. A single species of *Hepatozoon canis* has frequently been identified. This apicomplexan parasite is primarily transmitted by the brown tick *Rhipicephalus sanguineus* sensu lato [2, 3].

Infection in dogs is associated with regions characterized by tropical, subtropical, or temperate climates, where vector-borne transmission is sustained. The prevalence has been reported to range from 0.49% to 79% based on the geographical area, source of the dogs, and diagnostic methods [4, 5]. In South America, the disease has only been documented in Venezuela, Colombia, Brazil, Argentina, and Chile [6–8]. In domestic dogs, *H. canis* can manifest as a standalone condition or in conjunction with other hemopathogens. It can be opportunistic, asymptomatic, or present with mild-to-severe symptomatology [9]. Additionally, host-related factors, such as age, splenectomy, immune competence, and concomitant infections or diseases, should be considered [10].

The clinical presentation and hematological changes associated with hepatozoonosis are similar to those observed in other tick-borne diseases [11]. However, the therapeutic approach employed differs significantly, emphasizing the importance of prompt identification at the time of a suspected diagnosis [12, 13].

Although there are no official reports on clinical diseases in Peru, the presence of suitable environmental conditions for vector growth, particularly in the northern Lima region, indicates a potential risk. Favorable climatic conditions and immigration facilitate the transfer of transboundary diseases to neighboring territories [14]. Consequently, other vector-borne diseases such as ehrlichiosis, anaplasmosis, and canine babesiosis have been frequently detected in Peru [15, 16].

The aim of this study was to detect *H. canis* in domestic dogs exhibiting clinical signs and hematological changes in the northern area of Lima, Peru.

## 2. Materials and Methods

### 2.1. Study Location

The study was conducted in the districts of northern Lima, Peru, at an altitude of 200–300 m above sea level. The mean temperature during the summer months (December to March) is 30°C, whereas that during the winter months (July to September) is 15°C. Samples were collected between October 2021 and December 2022 from veterinary clinics in the northern Lima area, which has a high prevalence of tick-borne hemopathogens [16] and ticks [17].

### 2.2. Samples

A total of 152 dogs, aged 4 months or older, were included in the study, without distinction of breed or sex. The dogs displayed clinical signs and hematological alterations, including leukocytosis, anemia, or thrombocytopenia. Blood samples (2–4 mL) were obtained from each animal and divided into two tubes containing EDTA as an anticoagulant. One tube was used for hematological analysis, while the other was reserved for PCR testing. Automated complete blood counts and blood smears were performed. A blood smear from each sample was used to identify intracellular inclusions of *H. canis* or other hemopathogens through direct observation using an optical microscope.

### 2.3. PCR Analysis

A total of 200 *μ*L of blood was used for DNA extraction using the silica column extraction method (GeneJET DNA Purification Kit, Thermo Fisher Scientific, United States). The quality of the extracted DNA was assessed based on the 1.8–2.0 ratio at 260/280 nm using a NanoDrop spectrophotometer (Thermo Fisher Scientific, United States).

The primers used for the detection of a partial *Hepatozoon* sp. sequence were 18S-F: 5⁣′-GGTAATTCTAGAGCTAATACATGAGC-3⁣′ and 18S-R: 5⁣′-ACAATAAAGTAAAAAACAYTTCAAAG-3⁣′ [18]. The PCR master mix contained DNA from each sample (1 ng/*μ*L), 0.75 *μ*M of each primer (forward and reverse), 2.5 mM of MgCl_2_, 0.2 mM of dNTPs, 1.25 U/reaction and 1X PCR buffer of Platinum *Taq* DNA Polymerase (Invitrogen, United States), and nuclease-free water (Sigma-Aldrich, United States) in a final volume of 20 *μ*L. PCR cycling conditions (PTC-200 Thermal Cycler, MJ Research, United States) were as follows: polymerase activation at 94°C for 2 min, followed by 35 cycles of denaturation at 94°C for 30 s, annealing at 54°C for 30 s, and extension at 72°C for 45 s. The final extension step was performed at 72°C for 5 min [11].

A negative DNA control (healthy dog with no history of hemoparasitosis; negative serological test for *Anaplasma*, *Ehrlichia*, *Borrelia*, and *Dirofilaria*; and no history of ticks) was included. The positive control, *H. canis* DNA, was kindly provided by the Laboratory of Zoonotic and Vector-Borne Diseases, School of Veterinary Medicine, Hebrew University of Jerusalem, Israel.

Sanger sequencing (Macrogen, Korea) was conducted on 10 strong positive amplicons of 574 bp. The amplicons were sequenced in both directions using the same PCR primers. The resulting sequences (forward and reverse) of each amplicon were aligned using ClustalX, Version 2.1. The aligned sequences were then analyzed using BioEdit Version 7.2.5, where gaps were removed, and a corrected sequence was obtained manually. Subsequently, this sequence was compared with the GenBank database using the Basic Local Alignment Search Tool (BLAST) program (https://blast.ncbi.nlm.nih.gov/Blast.cgi). Finally, percentages of coverage, identity, and expected value (*E*) were recorded. Phylogenetic trees were constructed using the maximum likelihood (ML) method with 1000 bootstrap replicates using the MEGA 11 program [19]. The best-fitting nucleotide substitution model was selected based on the lowest value of the Bayesian information criterion (BIC) using the Kimura two-parameter (K2P) model [20].

### 2.4. Data Analysis

The results of the qualitative variables were summarized using absolute and relative frequency tables. The quantitative variables of age were summarized using measures of central tendency and dispersion.

### 2.5. Ethical Considerations

This study was evaluated and approved by the Institutional Ethics Committee for the Use of Animals of the Universidad Peruana Cayetano Heredia (Registration Code 201937, Approval Certificate 032-10-20).

## 3. Results

**Figure 1:**
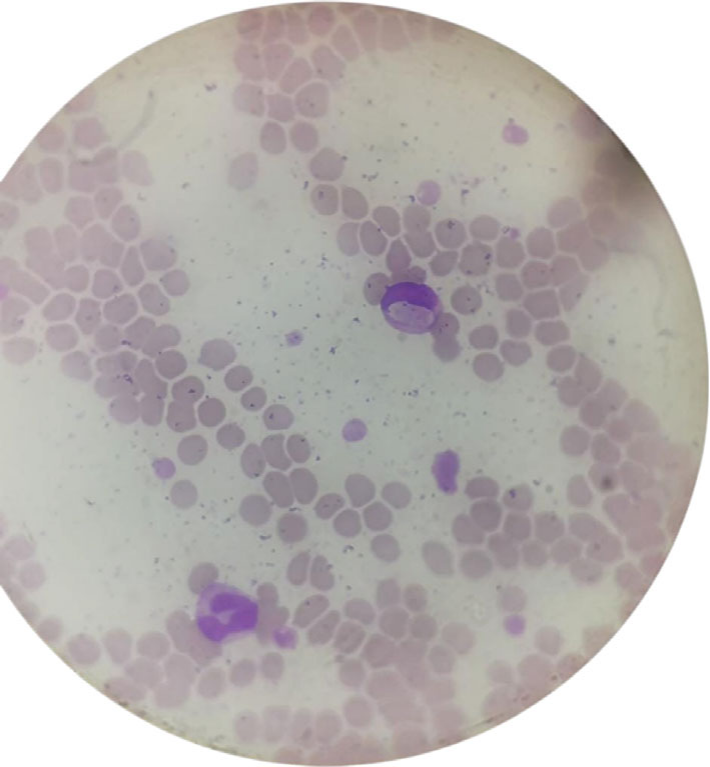
Blood smear of a dog with hematologic disorders. Presence of an oval inclusion body (gamont) of *H. canis* inside the cytoplasm of neutrophils. Wright's stain, original magnification ×100.

A total of 15.1% (23/152) of the dogs tested positive for *H. canis* by PCR, whereas 0.7% (1/152) tested positive by the blood smear technique (Figure 1).

The 18S rRNA sequence obtained from the amplicons (Accession Number PX061991) exhibited > 95% coverage and > 99% identity with the sequences in the GenBank database. The phylogenetic tree revealed a similarity between the *H. canis* sequences obtained in this study and those in Colombia (MK910141), Thailand (MK830996, KF621082, MZ151506), the Philippines (LC428208), Pakistan (KU535868), and India (MG050163). Nevertheless, these results were not significant because of the lower level of bootstraps obtained (less than 50).

The most frequent hematological alterations in *H. canis*-positive dogs were anemia (73.9%, 17/23), thrombocytopenia (56.5%, 13/23), and leukocytosis (52.2%, 12/23). Among the dogs with anemia, normocytic and normochromic anemias were the most frequent.

In terms of demographic characteristics, a greater proportion of positive cases was identified in male dogs (17.4%, 15/86) and seniors (28.6%, 6/21) (Table 1).

The most frequent clinical manifestations observed in *H. canis*-infected dogs were lethargy, anorexia, pallid mucous membranes, splenomegaly, and fever (Table 2).

Furthermore, *E. canis* morulae was identified in 1.3% (2/152) of samples that tested negative for *H. canis*. The 71.42% (10/14) of some selected samples were seropositive for *Ehrlichia* spp. and negative for *H. canis*. Among the *Ehrlichia* sp. seropositive animals, 50% (5/10) were coinfected with *H. canis*. Coinfection is characterized by lethargy, inappetence, splenomegaly, anemia, and thrombocytopenia.

## 4. Discussion

The presence of *H. canis*-positive dogs in the present study, along with other hemopathogens, can be attributed to the fact that Lima has conditions comparable to those of other cities where previous reports of the disease have been documented. Therefore, it is imperative to consider the role of the vector and the overpopulation of stray dogs as significant contributing factors. The frequency observed in this study was lower than that in neighboring countries such as Venezuela, Colombia, and Brazil. Furthermore, the prevalence of vectors in these countries may be higher because of their tropical climate. It is noteworthy that *Hepatozoon* is frequently underdetected in veterinary clinics in Peru, particularly compared to other diseases such as ehrlichiosis and anaplasmosis.

The frequency of positive results was higher in males, which may be attributed to their tendency to engage in vagrant behavior, as well as the fact that their population is larger than that of females [21]. Furthermore, senior dogs were the most frequently affected, which can be attributed to prolonged exposure to the vector, the presence of concomitant diseases, and the immunological status of the animal [22]. The frequency of positive results for age and sex was also consistent with findings from other countries, including Venezuela, Argentina, and Brazil [10, 23, 24].

PCR is the most widely used technique because of its high sensitivity and specificity [25]. In contrast, detection using the blood smear technique is affected by the level of parasitemia and the sampling time, which may coincide with the merogony of the parasite [10, 26]. Similar to our findings regarding the sensitivity of PCR, a study conducted in southeastern Brazil found that 43% of dogs tested positive for *H. canis* by PCR but negative by blood smear [24]. Although PCR requires the use of thermal cyclers and training, it can be replaced by molecular isothermal amplification techniques, which facilitate practical use at point-of-care (POC) in veterinary clinics without resorting to sophisticated laboratories [27, 28].

The *H. canis* sequences obtained in the present study were similar to those isolated from dogs in other regions of the world, including Colombia [29], Thailand [30], the Philippines [31], Pakistan [32], and India [33]. These findings reveal a close relationship between these pathogens distributed worldwide; it is interesting to observe their evolutionary and pathogenic characteristics, which may be associated with specific emerging genotypes [34, 35].

Northern Lima, Peru, is experiencing an overpopulation of pets and a high rate of stray dogs [21, 36]. In recent years, there has been an increase in the number of dogs entering Peru, including those from tropical countries that are endemic for hemoparasitosis [14]. These factors, along with the environmental and vector-related characteristics, may contribute to the development of endemic hepatozoonosis.

Timely diagnosis of a disease is crucial for implementing effective control measures. Toltrazuril is one of the most commonly used drugs in Latin America to treat hepatozoonosis and coccidiosis [37]. In light of the evidence presented, it is imperative that pharmaceutical companies in Peru are equipped to either produce or import specific drugs formulated at the appropriate concentrations and indications for the treatment of canine hepatozoonosis.

This study was limited by the absence of serological screening for other hemoparasites in all blood samples. Such an approach would have enabled the determination of other potential causes of infectious diseases and identification of coinfections. Similarly, noninfectious causes of hematological alterations were not considered. As a result of these limitations, it cannot be definitively concluded that *H. canis* is the sole causal agent of the observed clinical signs and hematological abnormalities. The clinical signs of hemoparasitosis are nonspecific, and due to the design of the study and the low number of positive animals, it was not possible to establish a relationship between the type of hematological alteration/clinical sign and positivity to *H. canis*. However, it has been suggested that the levels of leukocytes and neutrophils are elevated in comparison to other hemopathogens [38].

Furthermore, additional data regarding the provenance of dogs, such as information on travel to other locations within Peru or their country of origin, were not collected. It is noteworthy that the present study included dogs that originated from neighboring countries (Venezuela and Brazil). One dog tested positive for *H. canis*; the animal had resided in Venezuela for 12 years, whereas the other had resided in Peru for approximately 1 year.

The frequent detection of this agent based on the suspicion of hemoparasitosis represents a significant finding for veterinarians. It is recommended that veterinarians include hepatozoonosis in their differential diagnosis. It is also crucial to conduct epidemiological surveillance using appropriate tools, including molecular techniques. Laboratories may implement commercial *in vitro* diagnostic tests, as has already been performed for commercial POC tests for ehrlichiosis, anaplasmosis, and canine babesiosis.

Although the risk of zoonotic transmission appears to be low, given the single reported case of *Hepatozoon* spp. in a human with hematological disorders, caution should be exercised when handling dogs infested with ticks because other zoonotic pathogens may be transmitted [39].

In conclusion, *H. canis* was detected for the first time in an area of Peru, with a high prevalence of vector-borne diseases in dogs with clinical signs suggestive of hemoparasitosis.

## Figures and Tables

**Table 1:** Demographic characteristics of *H. canis*-positive dogs exhibiting clinical signs compatible with hemoparasitosis in northern Lima during 2021-2022 (*N* = 152).

**Demographic variable**	**Dogs**	***H. canis*-positive**	
	**N**	**n**	**%**
Sex			
Male	86	15	17.4
Female	66	8	12.1
Age			
Puppy	50	8	16.0
Adult	81	9	11.1
Senior	21	6	28.6
Housing district			
Puente Piedra	56	9	16.1
Ancón	23	3	13.0
Carabayllo	21	4	19.0
Santa Rosa	16	2	12.5
San Martín de Porres	13	1	7.7
Los Olivos	10	2	20.0
Comas	9	2	22.2
Independencia	4	0	0

**Table 2:** Clinical signs of *H. canis*-positive dogs compatible with hemoparasitosis in northern Lima during 2021-2022 (*N* = 152).

**Clinical signs**	**Dogs**	***H. canis*-positive**	
	**N**	**n**	**%**
Lethargy	88	16	18.2
Inappetence	87	14	16.1
Pale mucous membranes	68	11	16.2
Splenomegaly	52	9	17.3
Fever	51	7	13.7
Vomiting	24	6	25.0
Lymphadenomegaly	17	4	23.5
Cough	16	2	12.5
Dyspnea	12	3	25.0
Diarrhea	11	3	27.3
Bleeding	11	1	9.1
Lameness	9	3	33.3
Hepatomegaly	8	1	12.5
Cachexia	5	1	20.0
Petechiae	3	1	33.3
Jaundice	2	1	50.0
Ascites	1	1	100.0
Pneumonia	1	1	100.0

## Data Availability

The data that support the findings of this study are available from the corresponding author upon reasonable request.
